# Detection of VAMP Proteolysis by Tetanus and Botulinum Neurotoxin Type B In Vivo with a Cleavage-Specific Antibody

**DOI:** 10.3390/ijms23084355

**Published:** 2022-04-14

**Authors:** Federico Fabris, Petra Šoštarić, Ivica Matak, Thomas Binz, Anna Toffan, Morena Simonato, Cesare Montecucco, Marco Pirazzini, Ornella Rossetto

**Affiliations:** 1 Department of Biomedical Sciences, University of Padova, Via Ugo Bassi 58/B, 35131 Padova, Italy; federico.fabris.5@phd.unipd.it (F.F.); cesare.montecucco@gmail.com (C.M.); 2 Department of Pharmacology, School of Medicine, University of Zagreb, Šalata 11, 10000 Zagreb, Croatia; petra.sostaric@mef.hr (P.Š.); ivica.matak@mef.hr (I.M.); 3 Institute of Cellular Biochemistry, Hannover Medical School, Carl-Neuberg-Straße 1, 30625 Hannover, Germany; binz.thomas@mh-hannover.de; 4 Istituto Zooprofilattico Sperimentale delle Venezie, Viale dell’Università 10, 35020 Legnaro, Italy; atoffan@izsvenezie.it; 5 Institute of Neuroscience, Italian Research Council, University of Padova, Via Ugo Bassi 58/B, 35131 Padova, Italy; morena.simonato@cnr.it; 6 Interdepartmental Research Center of Myology CIR-Myo, University of Padova, Via Ugo Bassi 58/B, 35131 Padova, Italy

**Keywords:** botulinum neurotoxins, tetanus neurotoxins, vesicle-associated membrane protein VAMP, SNARE proteins, polyclonal antibodies

## Abstract

Tetanus and Botulinum type B neurotoxins are bacterial metalloproteases that specifically cleave the vesicle-associated membrane protein VAMP at an identical peptide bond, resulting in inhibition of neuroexocytosis. The minute amounts of these neurotoxins commonly used in experimental animals are not detectable, nor is detection of their VAMP substrate sensitive enough. The immune detection of the cleaved substrate is much more sensitive, as we have previously shown for botulinum neurotoxin type A. Here, we describe the production in rabbit of a polyclonal antibody raised versus a peptide encompassing the 13 residues C-terminal with respect to the neurotoxin cleavage site. The antibody was affinity purified and found to recognize, with high specificity and selectivity, the novel N-terminus of VAMP that becomes exposed after cleavage by tetanus toxin and botulinum toxin type B. This antibody recognizes the neoepitope not only in native and denatured VAMP but also in cultured neurons and in neurons in vivo in neurotoxin-treated mice or rats, suggesting the great potential of this novel tool to elucidate tetanus and botulinum B toxin activity in vivo.

## 1. Introduction

Tetanus (TeNT) and Botulinum Neurotoxins (BoNTs) form the large family of Clostridial Neurotoxins (CNT), the deadly bacterial exotoxins made by anaerobic and sporogenic Clostridium species [1]. *C. tetani* produces one single TeNT, whereas several phylogenetically distinct clostridia, including *C. botulinum*, *butyricum*, *baratii*, and *argentinense*, produce eight different BoNT serotypes. These toxins are indicated with an alphabetical letter (BoNT/A through/G and BoNT/X) and are further subclassified as subtypes (numerals appended to the serotype) [1,2,3].

CNT display lethal doses in the low ng/kg range, and thus are the most poisonous substances discovered so far [4]. This potency derives from the ability of CNT to enter selectively into nerve terminals and to cleave one (or more) neuronal SNARE (Soluble NSF Attachment protein Receptors) proteins, i.e., VAMP-1/2, SNAP-25 or syntaxin-1A/1B. These three proteins form the heterotrimeric complex, known as the SNARE complex, which is responsible for synaptic vesicle fusion with the presynaptic plasma membrane and neurotransmitter release [5,6]. SNARE cleavage by CNT prevents the formation of the complex or causes its dysfunction, thereby inhibiting neurotransmission.

BoNTs bind to and enter within both motor and autonomic peripheral cholinergic neurons, where they block the release of the neurotransmitter acetylcholine, causing the flaccid paralysis of botulism [1,7]. Accordingly, minute amounts of BoNT/A1 are used, with great success and safety, to treat neurological conditions caused by hyperactivity of nerve terminals and in aesthetic medicine [8]. Conversely, TeNT undergoes a retroaxonal transport and it is released within the spinal cord and brain stem, where it blocks neurotransmission of inhibitory interneurons causing excitatory disbalance and spastic paralysis, which is the main symptom of tetanus [9,10,11]. Tetanus can be prevented by a very effective vaccine [9,11], whereas no vaccine has yet been approved for BoNTs, perhaps due to the rarity of botulism and the extensive use of BoNT/A1 in human therapy [12,13].

The metalloprotease activity of CNT against SNARE proteins is sufficient to cause the cardinal symptoms of tetanus and botulism. The identification of VAMP-1/2 as the targets of TeNT and BoNT/B [14] rapidly led to the discovery of the other BoNT substrates, i.e., SNAP-25 for BoNT/A, BoNT/C, and BoNT/E [15,16], and VAMP-1/2 for BoNT/D, BoNT/F, and BoNT/G [17,18,19]. BoNT/C is unique as it also cleaves syntaxin-1A/1B [20], although its toxic effect is primarily due to SNAP-25 proteolysis [21].

Different experimental techniques have been developed to track SNARE cleavage as a readout to monitor CNT activity in cell and neuronal culture [22,23,24,25,26,27,28,29,30], in animal tissues [21,31,32], as well as for CNT detection in biological isolates [33,34,35,36,37,38]. A particularly convenient and versatile method is the use of antibodies against specific portions of VAMP, SNAP-25, or syntaxin, which can be used in cell-based assays via Western blotting [22,24,26,28,39,40,41,42], and by fluorescence microscopy [21,29,31,32,43,44,45,46,47,48,49,50,51,52].

Depending on the specific antigen used to elicit host immunization, different antibodies recognizing different portions of the SNARE proteins have been developed and detect CNT proteolytic activity very specifically. Some antibodies recognize the intact SNARE protein, and their signal decreases after cleavage [53,54,55]. Other antibodies recognize both the entire and the truncated form of the SNARE protein and are useful to evaluate CNT activity by Western blotting in a ratiometric manner [21,24,40,56,57,58,59,60,61]. Particularly useful are those antibodies recognizing the SNARE protein only after cleavage by a specific toxin because of the development of in vitro detection assays [62,63,64,65] and to track CNT activity in vivo, although this is thus far restricted to the BoNT/A- or BoNT/E-truncated forms of SNAP-25 [21,31,32,43,44,45,46,47,48,49,50,51,52,66,67,68]. These antibodies have the advantage of providing a signal that increases in parallel with the progressive CNT proteolytic activity in tissues where it is ineffective, monitoring a decreasing signal.

Here, we describe the development of a polyclonal antibody specific for segment 77-89 of mouse VAMP-2 (dubbed Ab-VAMP_77_), which selectively recognizes VAMP-1/2 only after TeNT and BoNT/B cleavage that occurs at the same single site [8,14]. Notably, Ab-VAMP_77_ detects the proteolytic activity of TeNT and BoNT/B, but not the one elicited by BoNT/D or BoNT/G, in neuronal cultures. More importantly, it raises a robust signal in motor axon terminals at the neuromuscular junction (NMJ) after BoNT/B injection and in the brain stem after injection of TeNT. Ab-VAMP_77_ represents a novel and effective tool to visualize the proteolytic activity of these two toxins in vitro and in vivo.

## 2. Results and Discussion

### 2.1. Generation of the Polyclonal Antibody Specific for TeNT and BoNT/B Cleavage of VAMP

**Figure 1:**
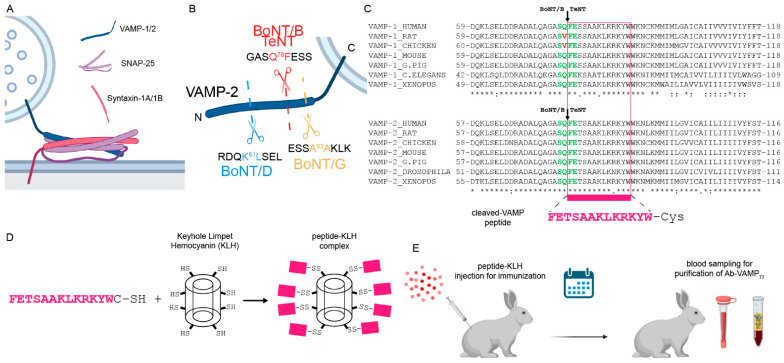
Generation of the Ab-VAMP_77_ antibody. (**A**) Scheme showing the three SNARE proteins involved in neuroexocytosis that form the SNARE complex by coil-coiling their SNARE motifs, one from the vesicular VAMP-1/2 (blue), two from the membrane-anchored SNAP-25 (pink), and one from the integral membrane protein syntaxin-1A/1B (red). (**B**) Scheme showing the peptide bonds cleaved by the VAMP-specific CNT used in this study, which generate specific new N-termini to VAMP (shown is human VAMP-2). (**C**) Alignment of VAMP-1/2 showing that the FETSAAKLKRKYW peptide (pink) exposed by BoNT/B and TeNT is in all the main animal species used in research. The green residues indicate cleavage sites of TeNT and BoNT/B in VAMP-1 and VAMP-2. Red residues indicate the mutation responsible for VAMP-1 resistance to TeNT and BoNT/B in rat and chicken. (**D**) Scheme showing the generation of immunogenic carrier by chemical conjugation of the C-terminal Cysteine to the Keyhole limpet Hemocyanin. (**E**) the peptide-KLH complex was injected into a rabbit, and at the scheduled time point the blood was collected for the ensuing purification of peptide-specific IgGs.

VAMP-1 and -2 are integral proteins with a single transmembrane domain spanning the synaptic vesicle membrane [69,70]. VAMP-1 and VAMP-2 are protein isoforms differing significantly only in the 20 N-terminal residues, and for the remaining part are highly conserved among vertebrate and invertebrate animals [71] (Supplementary Figure S1). This cytosolic portion contains the alpha-helical domain (SNARE motif) that coil-coils with two similar domains of SNAP-25 and one of syntaxin to form the SNARE complex involved in neuroexocytosis (Figure 1A) [5]. All the CNT targeting VAMP cleave single and specific peptide bonds within the SNARE-motif (Figure 1B). TeNT and BoNT/B cleave the identical peptide bond Q78F79 in VAMP-1 and Q76F77 in VAMP-2 (with human VAMP numbering), leaving the truncated form of VAMP on the SV membrane. Notably, due to the high conservation of the two proteins, the same truncated VAMP-1 and VAMP-2 are expected to be generated in all the animal species used in the lab except for the rat and chicken VAMP-1, which are resistant to TeNT and BoNT/B cleavage [8,72] (Figure 1C).

An antibody specific to BoNT/B and TeNT cleaved VAMP, but not to the intact protein, is an invaluable tool for visualizing the action of these toxins in vivo. For this purpose, the peptide FETSAAKLKRKYW-C corresponding to the N-terminus of cleaved VAMP-2 bearing a C-terminal Cysteine was conjugated to Keyhole limpet hemocyanin, used as an immunogenic carrier (Figure 1D,E). The rabbit was immunized and, after boosting injections, scheduled blood samplings were undertaken. Sera were eventually pooled and used for affinity purification. To this end, the C-terminal Cysteine was replaced by biotin for a fast coupling to a streptavidin-agarose resin, which allowed a very fast and efficient affinity purification of the antibody (Supplementary Figure S2).

### 2.2. Ab-VAMP_77_ Detects, with High Specificity, TeNT and BoNT/B Cleavage in Primary Neuronal Cultures

Neuronal cultures are widely employed to dissect the mechanism of action of CNTs. In addition, after the identification of SNARE proteins as specific targets of CNTs, TeNT and BoNTs became major tools to study the exo–endocytosis apparatus within neurons, as well as other cell types [8,73]. Moreover, neuronal cultures are used as a screening platform to assess potential CNT inhibitors [12].

**Figure 2:**
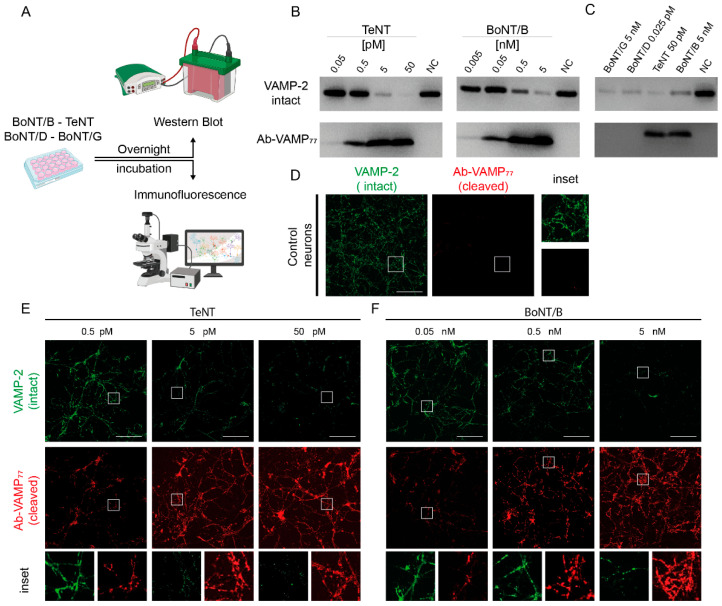
The Ab-VAMP_77_ polyclonal antibody detects VAMP-1/2 cleavage by TeNT and BoNT/B in neuronal culture with high efficiency and toxin selectivity. (**A**) Scheme showing the experimental readout used to evaluate the specificity of Ab-VAMP_77_ in rat CGNs. (**B**) Representative Western blotting showing the high sensitivity of Ab-VAMP_77_ compared to a conventional antibody at low TeNT (**left**) and BoNT/B (**right**) concentrations. The NC samples are control neurons not treated with any toxin. (**C**) Representative Western blotting showing that Ab-VAMP_77_ binds only to a cleaved fragment of VAMP specifically generated by BoNT/B and TeNT, but not by BoNT/D nor BoNT/G. (**D**) Control CGNs showing the typical punctuate pattern (inset) of VAMP-2 (green) staining at presynaptic level, and no signal for Ab-VAMP_77_ in the absence of TeNT or BoNT/B treatment. (**E**,**F**) Immunofluorescence analysis showing that at increasing concentration of TeNT (**E**) or BoNT/B (**F**) the signal of intact VAMP-2 (green) fades away, whereas the signal of Ab-VAMP_77_ (red) progressively increases yet maintains the typical punctuate pattern (insets) in both cases. Scale bars in (**D**–**F**) are 100 µm.

A very convenient model to assay the specificity of antibodies in detecting cleavage by TeNT and BoNT/B is Cerebellar Granule Neurons (CGNs), a primary neuronal culture that is highly susceptible to CNTs [68]. We thus intoxicated CGNs for 12 h with four CNTs targeting VAMP: TeNT, BoNT/B, BoNT/D, and BoNT/G, and monitored their protease activity via Western blotting and imaging (Figure 2A). The proteolytic activity of TeNT can be monitored with a VAMP-2 specific commercial antibody that provides a decreasing signal upon cleavage. As shown in Figure 2B, 50 pMolar TeNT causes the complete disappearance of VAMP-2, with 5 pMolar being almost equipotent. At variance, 0.5 and 0.05 pMolar TeNT do not apparently cause a decrease in the signal, indicating that these two concentrations of TeNT are not sufficient to mediate cleavage of VAMP-2. However, when the same samples were stained with Ab-VAMP_77_, a clear band appeared at 0.5 pMolar and a fainter band was still visible in the 0.05 pMolar sample. This result indicates that Ab-VAMP_77_, by providing a rising rather than a decreasing signal, is much more sensitive than anti-intact VAMP antibody when detecting the TeNT-mediated VAMP-2 proteolysis. The same enhanced sensitivity is also observed after VAMP-2 cleavage by BoNT/B (Figure 2B). Intact VAMP is not recognized Ab-VAMP_77_, indicating its selectivity only for cleaved VAMP. This is of fundamental importance for in vivo studies.

We next tested Ab-VAMP_77_ in neurons treated with BoNT/D or BoNT/G, as they generate two different cleaved-VAMP N-termini containing or not the linear epitope. In fact, BoNT/D cleaves peptide bond K61L62 in VAMP-1, K59L60 in VAMP-2 (human VAMP numbering), upstream of the TeNT-BoNT/B cleavage site and BoNT/G cleaves the A83A84 bond in VAMP-1, A81A82 in VAMP-2 (human numbering) included in the antigenic peptide (Supplementary Figure S3A). BoNT/D is very potent in CGNs and cleaved all the VAMP-2 already at fMolar concentration (Supplementary Figure S3B), while BoNT/G has an activity more similar to BoNT/B and thus required a nMolar range for complete cleavage (Supplementary Figure S3C). In both cases, Ab-VAMP_77_ did not recognize the truncated VAMPs at any toxin concentration (Supplementary Figure S3B,C), including those causing complete cleavage (Figure 2C).

Comparable results were obtained when VAMP-2 cleavage was monitored by immunostaining. As shown in Figure 2D, control CGNs stained with the antibody against the whole protein displayed the typical punctuate accumulation of VAMP-2 at the level of presynaptic boutons, whilst Ab-VAMP_77_ did not produce any detectable signal. When treated with increasing concentrations of TeNT and BoNT/B, the signal of intact VAMP-2 progressively faded away (Figure 2E,F), and eventually disappeared almost completely. The antibody against cleaved VAMP displayed the opposite pattern, i.e., it increased proportionally to the concentration of TeNT and BoNT/B. As shown in the insets, the signal remained almost exclusively confined at the level of the puncta, coherently with previous observations reporting that after SNARE cleavage by CNT, synaptic vesicles accumulate at the presynaptic level [22,74,75]. Notably, when the signal given by Ab-VAMP_77_ was merged with the signal of the residual intact VAMP, the two signals displayed clearly segregated staining (Supplementary Figure S4). This is consistent with the fact that the two antibodies spot distinct and mutually exclusive populations of VAMP-2 corresponding to the intact and the cleaved one. Again, in spite of a complete cleavage of VAMP-2, Ab-VAMP_77_ was unable to detect VAMP cleavage products after BoNT/D and BoNT/G treatment (Supplementary Figure S3D,E).

Altogether, these results indicate that Ab-VAMP_77_ can detect the cleavage of VAMP by TeNT and BoNT/B with high specificity and selectivity, which are maintained both when VAMP is denatured as well as when the epitope is fixed and in native conformation, consistent with the fact that the antibody was raised with a linear peptide and that the epitope is sequential rather than conformational. Ab-VAMP_77_ does not bind intact VAMP. At variance, Gray et al. developed a monoclonal antibody using the same FETSAAKLKRKYW peptide that recognizes TeNT-BoNT/B-cleaved VAMP, but also the intact protein [64]. At the same time, our finding is in agreement with the results obtained by von Berg et al., who developed a monoclonal antibody against a similar, yet shorter, peptide (FETSAAKL) that recognized specifically the cleaved, but not the intact form of VAMP [35]. It is tempting to suggest that our immunization protocol has favored an immune response specific to the very N-terminus of the peptide, raising antibodies that, similarly to that of von Berg et al., are specific to the TeNT-BoNT/B newly generated epitope in VAMP. At variance, Gray et al. might have selected a monoclonal antibody recognizing another part of the peptide, which remains exposed for binding both in intact and TeNT-BoNT/B-cleaved VAMP. Concurrently with this possibility, the Gray et al. antibody fails to detect VAMP after BoNT/G cleavage [64].

### 2.3. The Antibody against Cleaved VAMP Specifically Detects the Activity of BoNT/B at the Neuromuscular Junction

**Figure 3:**
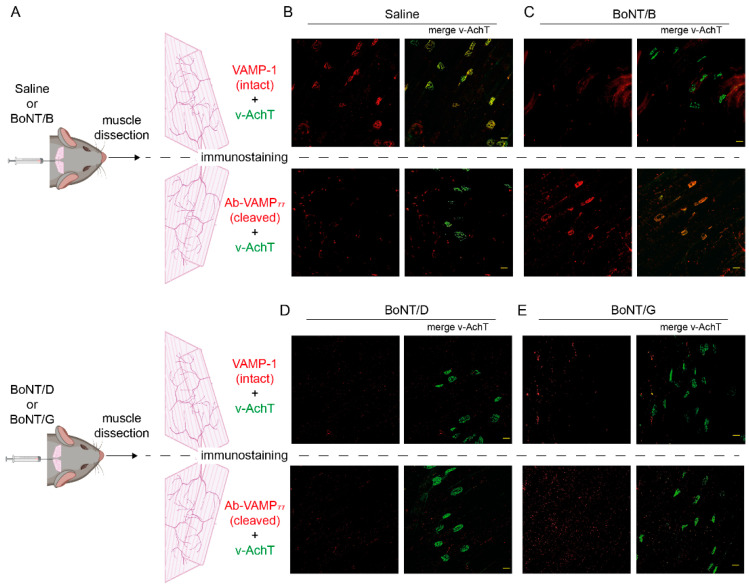
The Ab-VAMP_77_ polyclonal antibody detects VAMP cleavage by BoNT/B at the neuromuscular junction. (**A**) Scheme showing the staining of the two LAL muscles collected after injection of either saline or BoNT/B (top panel) or of either BoNT/D or BoNT/G (bottom panel) and the following immunostaining of one LAL with an antibody against intact VAMP-1 (top panels) or with the Ab-VAMP_77_ antibody (bottom panels). In both cases, the muscles were also stained for the vesicular protein marker v-AchT. (**B**–**E**) Representative images of whole-mount LAL muscles injected with either saline (**B**) or BoNT/B (**C**) or BoNT/D (**D**) or BoNT/G (**E**) stained for intact VAMP-1 (red in top panels) or cleaved VAMP (red in bottom panels). The right panels of each condition show the merge with v-AchT (green). Scale bar = 25 μm.

We next tested whether Ab-VAMP_77_ can detect BoNT/B activity in vivo within motor axon terminals at the NMJ. For this purpose, we injected a sub-lethal amount of BoNT/B (or saline as a control) between the ears of mice to provide a local intoxication of the Levator Auris Longus (LAL) muscles. LAL muscles originate from the cranial midline and extend toward the external ears, and consist of a few layers of myofibers making them very convenient for NMJ imaging [76,77,78]. After 48 h from the injection, we collected the two LAL and stained one muscle with an antibody recognizing the intact form of VAMP-1, i.e., the major VAMP form expressed at the adult NMJ [55], and the other one with Ab-VAMP_77_ (Figure 3A). Both samples were also stained with an antibody against the vesicular Acetylcholine Transporter (v-AchT), used as a marker of synaptic vesicles. As expected, LAL muscles injected with saline displayed clear presynaptic staining for intact VAMP-1 that colocalized with v-AchT, but no signal for cleaved-VAMP, indicating that Ab-VAMP_77_ does not bind intact VAMP-1 in vivo (Figure 3B). Conversely, the LAL muscles injected with BoNT/B displayed very bright and robust staining only with the antibody against cleaved-VAMP and only at the presynaptic level where it completely colocalized with v-AchT (Figure 3C), consistent with the fact that the antibody recognized an epitope associated with the synaptic vesicles. In keeping with the results on cultured CGNs, Ab-VAMP_77_ failed to recognize VAMP proteolysis operated by both BoNT/D (Figure 3D) and BoNT/G (Figure 3E). Importantly, it also failed to detect BoNT/B activity when injected in rats that, like chickens, express a VAMP-1 resistant to BoNT/B owing to a point mutation at the toxin cleavage site [72] (Supplementary Figure S5). This control experiment, based on a natural knock-in, further proves the high selectivity of Ab-VAMP_77_ for BoNT/B proteolytic activity in vivo, which is also maintained when the epitope is in its native conformation.

### 2.4. The Antibody against Cleaved-VAMP Specifically Detects the Activity of TeNT in the Central Nervous System

**Figure 4:**
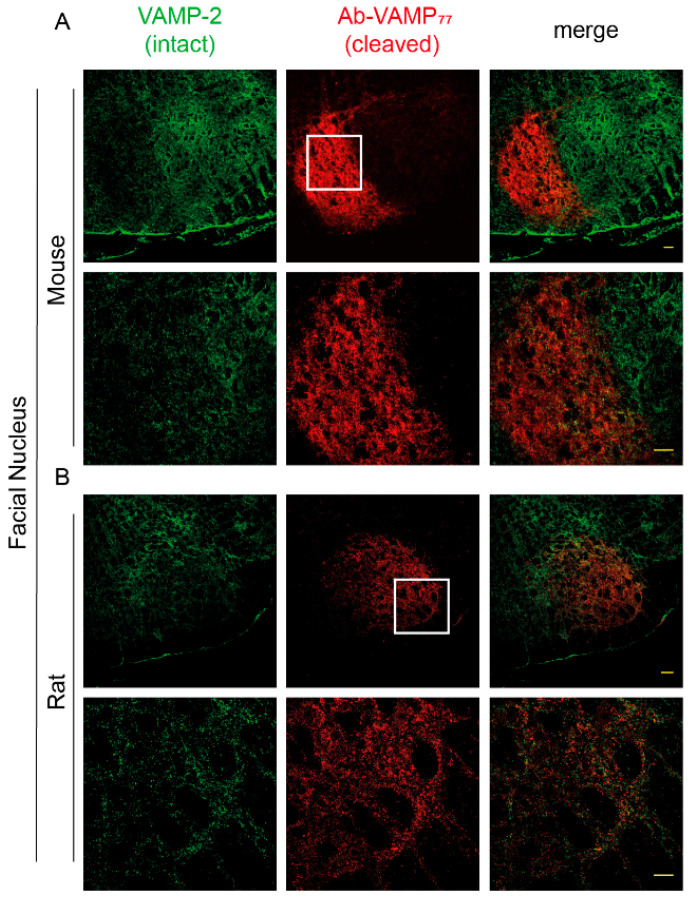
The Ab-VAMP_77_ polyclonal antibody detects VAMP cleavage by TeNT in the brain stem. The TeNT was injected either at the level of the LAL in the mouse (**A**) or at the level of the whisker pad in rats (**B**) and then the cleavage of VAMP was evaluated in the brain stem with an antibody against intact VAMP-2 (green) and Ab-VAMP_77_ (red). The bottom panels in (**A**,**B**) show a magnification of the white-squared area. Scale bar = 40 μm.

The strategy to exploit the protease activity as an amplifying factor to track down the tissue distribution of CNT has been instrumental in showing that BoNT/A action also acts in the CNS [79,80,81]. Notably, an antibody recognizing the BoNT/A-cleaved SNAP-25 was widely used to show that, upon intramuscular or subcutaneous injection, the toxin moves from the injection site via intra-axonal retrograde transport, reaching different areas of the CNS in an active form [43,44,45,47,48,50] even two synapses away from the injection site [46]. Therefore, we decided to test whether Ab-VAMP_77_ can detect the cleavage of VAMP via TeNT in the CNS. We opted for a model of local tetanus generated by injecting a sub-lethal amount of TeNT at the level of the LAL muscles, thereby causing a spastic paralysis confined to the muscles of the ears [11]. Two days after injection, we collected the brain stem and made cryoslices of the facial nucleus. As shown in Figure 4A, Ab-VAMP_77_ raised a very strong and bright, yet extremely confined staining for cleaved VAMP at the level of the seven dorsomedial and seven ventromedial subnuclei (7DM and 7VM) [82], the areas populated by the motor efferents innervating the LAL muscles. Of note, as shown by the magnification, in this case the signal also appeared as rounded varicosities, once again consistent with staining of presynaptic puncta. Moreover, it was restricted to areas deprived of the staining of intact VAMP-2 with little, if any, colocalization, again indicating that the two antibodies specifically recognize two distinct populations of VAMP. The same experiment performed in rats gave a similar result, although in this case the toxin was injected in the whisker pad and, accordingly, the signal appeared in a different area of the facial nucleus, i.e., the seven lateral and the seven dorsolateral subnuclei (7L and 7DL) [82], where the motor neurons commanding the muscles responsible for whiskers’ movements reside. Notably, in this case the signal also appeared less intense (Figure 4B), probably due to an overall reduced activity of TeNT compared to mice.

## 3. Conclusions

Clostridial neurotoxins are biochemical scalpels that surgically block neurotransmission at nerve terminals [8,83,84]. Not only the whole toxins, but also their isolated catalytic domains that are genetically expressed, transfected, or microinjected within target cells have been widely used to study the role of specific SNARE proteins in membrane fusion events, in neurons, in other cell types, and even in animal hosts naturally unsusceptible to their uptake [85,86,87,88,89]. Their use associated with modern biotechnologies still represents a reliable, efficient, and definitely convenient method to block neuroexocytosis in several biological systems [90,91]. Among them, TeNT and BoNT/B and their derivatives, which cleave the identical peptide bond in VAMP-1/2/3, are widely used. Here, we generated a polyclonal antibody recognizing the cleaved form of VAMP in a very specific and sensitive manner. This novel biochemical tool can be used both in Western blot and in immunofluorescence on cultured neurons, which we feel safe to extend to virtually any cell type TeNT or BoNT/B can be used on. This antibody can thus be used to image the in vivo proteolytic activity of TeNT and BoNT/B with high specificity using fluorescence microscopy. Compared to a recent report describing the properties of antibodies generated against the same linear epitope used here [64], Ab-VAMP_77_ does not bind intact VAMP and recognizes, in a very selective manner, the cleaved VAMP generated by TeNT and BoNT/B, but not those generated by other VAMP-cleaving toxins. Such specificity was also reported with a monoclonal antibody used to develop in vitro cleavage-based immunoassays to detect CNT in biological isolates [35], but our work led to the development of an antibody suitable for the detection of VAMP cleavage by BoNT/B at the neuromuscular junction and by TeNT in the central nervous system. This paves the way to future studies aimed at elucidating the action of these two toxins (or of their catalytic subunits) in vivo that was thus far not possible.

## 4. Materials and Methods

### 4.1. Antibodies, Toxins and Reagents

Botulinum Neurotoxins type B, D, and G were produced in *E. coli* (strain M15pREP4) as fusion proteins with a C-terminal StrepTag and purified on StrepTactin-Superflow matrix (IBA GmbH, Gottingen, Germany) as previously described [21,92]. Tetanus Neurotoxin was purified from C. tetani cultures [93]. Toxins were kept at −80 °C and diluted in complete culture medium for physiological solution plus 0.2% gelatin prior to use. Primary antibodies: anti-VAMP_77_ was produced in rabbit in this study (see below); anti-VAMP-1 was produced in our laboratory as previously described [32,94]; anti-v-AChT (guinea pig polyclonal 139 105) and anti-VAMP-2 (mouse monoclonal 104 211) were from Synaptic System (Gottingen, Germany). Secondary antibodies for immunofluorescence (anti-mouse, antirabbit, anti-guinea pig) conjugated to Alexa fluorophores were from Thermo Fisher Scientific (Waltham, MA, USA). Secondary antibodies for Western blotting (anti-mouse, antirabbit) conjugated to HRP were from Calbiochem (San Diego, CA, USA). Where not indicated, reagents were purchased from Sigma Aldrich (St. Louis, MO, USA).

### 4.2. Anticleaved-VAMP Antibody Production and Purification

A New Zealand white rabbit was immunized by subcutaneous injection with the peptide FETSAAKLKRKYWC coupled to KLH [38]. This peptide corresponds to amino acids 77-89 of mouse VAMP-2 with an additional C-terminal cysteine to link the peptide to KLH. Following the primary subcutaneous immunization on day 0, booster intra-muscular injections were performed on days 32 and 60. For each injection, 500 µg of KLH-peptide conjugate were mixed with the non-mineral oil-based adjuvant Montanide^TM^ ISA 763 VG (Seppic, Cedex, France). Rabbit serum was collected on day 120, frozen in liquid nitrogen, and stored at −80 °C until antibody purification.

For purification, 5 mg of peptide FETSAAKLKRKYK-(biotin)-NH_2_ (Caslo, Copenhagen, Denmark) was mixed with 500 µL of an agarose resin conjugated with Streptavidin (Thermo Fisher Scientific, cat. 20,359) and incubated into a disposable polypropylene column (cat. 29,922 from Pierce, Rockford, IL, USA) overnight at 4 °C in agitation for peptide–biotin–streptavidin coupling. The next day, the resin was extensively rinsed with PBS and then incubated (overnight at 4 °C) with the immune serum (previously ultracentrifuged for 15 min at 40,000 rpm at 4 °C to eliminate fat and blood cell debris). After overnight incubation, the resin was washed with 10 volumes of PBS. The antibodies were eventually eluted by the addition of 10 volumes (250 uL each) of glycine 0.1 M, pH 3.0. These fractions were collected in tubes containing 50 µL of Tris 1 M pH 7.4 to buffer the glycine solution. Protein concentration was assessed with Nanodrop (Thermo Fisher Scientific). Aliquots were then stored at −80 °C until use.

### 4.3. Cerebellar Granules Neurons Cultures

CGNs were prepared from 4–5-day-old rat pups as described in [68]. Cerebella were collected, mechanically disrupted, and enzymatically dissociated with trypsin in presence of DNase I. Cells were then plated in precoated (poly-l-lysine, 50 μg/mL) plastic 24 well plates or cover glasses at a cell density of 4 × 10^5^ or 2 × 10^5^ cells per well, respectively. Cultures were grown for at least 6 days at 37 °C, 5% CO_2_, BME supplemented with 10% fetal bovine serum, 25 mM KCl, 2 mM glutamine, and 50 μg/mL gentamicin. To block the proliferation of non-neuronal cells, cytosine arabinoside (10 μM) was added to the culture medium 18–24 h after plating.

### 4.4. Intoxication of CGNs with CNT

Seven days after CGNs preparation, cells were treated with indicated doses of either BoNT/B, BoNT/D, BoNT/G, or TeNT for 12 h in a complete culture medium. Cells plated on plastic were then directly lysed on the wells with Laemmli Sample Buffer (LSB) (Hepes 10 mM, NaCl 150 mM, SDS 1%, EDTA 4 mM, protease and phosphatase inhibitors) supplemented with mercaptoethanol and bromophenol blue, and collected for Western Blot analysis. Cells lysed in LSB were loaded onto NuPage 4–12% Bis-Tris gels for SDS-PAGE electrophoresis in MOPS buffer (Thermo Fisher Scientific, B0001) and then proteins were transferred onto Protran nitrocellulose membranes and saturated for 1 h in PBS-T (PBS, 0.1% Tween 20) supplemented with 5% non-fat dried milk (PanReac Applichem GmbH, Darmstadt, Germany). Incubation with primary antibodies (intact VAMP-2 1:2000; Ab-VAMP_77_ 1:2000) was performed overnight at 4 °C. Membranes were then washed with PBS-T and incubated at 4 °C with the appropriate HRP-conjugated secondary antibodies (1:5000) for 90 min. After extensive washings, signals were revealed with Luminata^TM^ using an Uvitec gel doc system (Uvitec, Cambridge, UK).

CGNs plated on cover glasses were washed with PBS and then fixed in 4% PFA in PBS for 15 min at room temperature for immunofluorescence analysis. PFA was quenched in 50 mM NH_4_Cl PBS for 15 min. Neurons were then incubated for 3 min using 0.5% Triton X-100 in PBS for membrane permeabilization. Thereafter, cells were washed with PBS followed by 1 h of saturation (0.5% BSA in PBS) and incubated with primary antibodies (intact VAMP-2 1:200; VAMP_77_ 1:200 diluted in PBS with 3% BSA) overnight at 4 °C. CGNs were then washed with PBS three times and incubated for 2 h at room temperature with appropriate secondary antibodies (1:200 diluted in PBS 3% BSA). After extensive washes with PBS, coverslips were finally mounted using Mounting Medium (Dako; Santa Clara, CA; USA) for fluorescence microscopy inspection.

### 4.5. Intramuscular Injection of BoNTs and TeNT

CD1 mice weighing 25–30 g were anesthetized with gaseous isoflurane and then injected with a sublethal dose of either BoNT/B (0.2 ng/kg) or BoNT/D (0.01 ng/kg) or BoNT/G (5 ng/kg) or TeNT (2 ng/kg), or vehicle (0.9% NaCl with 0.2% gelatin) at the level of the neck between the LAL muscles. Animals were then checked every 4 h. Mice treated with BoNTs were directly euthanized by cervical dislocation. The LAL muscles were directly collected and immediately fixed in 4% PFA for 30 min at room temperature. TeNT-treated mice were instead intracardiac perfused with PBS and immediately after with 4% PFA, and the brain stem was then collected and post-fixed for 1 h.

For rat experiments, male Wistar Han rats (University of Zagreb School of Medicine, Zagreb, Croatia), 3–4 months old, 350–450 g weights were anesthetized with ketamine/xylazine (70/7 mg·kg^−1^ i.p.), and then injected with a sublethal dose of BoNT/B (1 ng/kg) at the level of the gastrocnemius, or TeNT (0.1 ng/kg) in right whisker pad. The contralateral muscles were instead injected with vehicle (0.9% NaCl with saline 0.2% gelatin). After 48 h (BoNT/B) or 7 days (TeNT), rats were intracardiac perfused with PBS and immediately after with 4% PFA in PBS. Brain stems and solei were dissected and post-fixed for 1 h in 4% PFA in PBS.

LAL muscles from mice were used as a whole-mount preparation while the gastrocnemius from rats was separated in bundles of 10–20 myofibers under a dissection microscope.

For immunofluorescence staining, LAL and gastrocnemius muscle bundles were washed with PBS and quenched in 50 mM NH_4_Cl in PBS for 20 min, then permeabilized and saturated in blocking solution (15% goat serum, 2% BSA, 0.25% gelatine, 0.20% glycine, 0.5% Triton X-100 in PBS) for 90 min. The muscles were then incubated with primary antibodies (v-AchT 1:500; intact VAMP-1 1:500; VAMP_77_ 1:500) in blocking solution for 72 h at 4 °C, washed three times in PBS, and incubated with appropriate secondary antibodies (1:200 diluted in PBS 0.5% Triton X-100) for 2 h at room temperature. After extensive washes with PBS, the whole LAL and gastrocnemius bundles were mounted on coverslips using Mounting Medium (Dako) for fluorescence microscopy inspection.

Brain stems were frozen in OCT compound (Sakura Finetek, Torrance, CA, USA), cryosliced in 30 µm slices and left to dry at least for 12 h. Then, they were quenched in 50 mM NH_4_Cl in PBS for 20 min, saturated and permeabilized for 1 h in blocking solution, and then added to primary antibodies (intact VAMP-2 1:500; VAMP_77_ 1:500) in blocking solution overnight at 4 °C. Slices were then extensively washed and incubated with appropriate secondary antibodies (1:200 diluted in PBS 0.5% Triton X-100) for 90 min at room temperature. After extensive washes with PBS, slices were mounted on coverslips using Mounting Medium (Dako) for fluorescence microscopy inspection.

### 4.6. Microscopy

Samples from both neuronal culture and in vivo experiments were analyzed with a Leica SP5 Confocal microscope with a 40× HCX PL APO NA 1.4 oil immersion objective. Laser excitation line, power intensity, and emission range were chosen to minimize signal bleed-through. Laser excitation line, power intensity, and emission range were chosen according to each fluorophore in different samples to minimize bleed-through. Raw images were processed with ImageJ without altering the intensity of the signals.

## Data Availability

All the data are contained in the article.

## Supplementary Materials

The following supporting information can be downloaded at: https://www.mdpi.com/article/10.3390/ijms23084355/s1.
