# Supplementary material for: Detection of VAMP Proteolysis by Tetanus and Botulinum Neurotoxin Type B In Vivo with a Cleavage-Specific Antibody
Source: Int J Mol Sci. 2022 Apr 14;23(8):4355. doi: 10.3390/ijms23084355 (PMC9024618; doi:10.3390/ijms23084355)
Supplement: Supplementary file 1 [file ijms-23-04355-s001.zip › ijms-1670358-supplementary.pdf]

```
VAMP-1_HUMAN        1   MSAPAQPPAE-GTEGTAPGGGPPGPPPNMTSNRRLQQTQAQVEEVVDIIRVNVDKVLERDQKL 62
VAMP-1_RAT          1   MSAPAQPPAE-GTEGAAPGGGPPGPPPNTTSNRRLQQTQAQVEEVVDIMRVNVDKVLERDQKL 62
VAMP-1_CHICKEN      1   MSEPAQQPAPGAPEGGAPAGGPPGPPPNLSSNRRLQQTQAQVEEVVDIMRVNVDKVLERDQKL 63
VAMP-1_MOUSE        1   MSAPAQPPAE-GTEGAAPGGGPPGPPPNMTSNRRLQQTQAQVEEVVDIMRVNVDKVLERDQKL 62
VAMP-1_G.PIG        1   MSAPAQPPAE-GTEGAAPGGGPPGPPPNMTSNRRLQQTQAQVEEVVDIMRVNVDKVLERDQKL 62
VAMP-1_C.ELEGANS    1   --------MDAQGDAGAQGGSQGGP---RPSNKRLQQTQAQVDEVVGIMKVNVEKVLERDQKL 45
VAMP-1_XENOPUS      1   --------------MSTPGTS----ATGDPGNRRLQQTQAQVDEVVDIMRVNVDKVLERDQKL 52
                                        : . .         .*:*********:***.*::***:*********

VAMP-1_HUMAN        63  SELDDRADALQAGASQFESSAAKLKRKYWWKNCKMMIMLGAICAIIVVVIVIYFFT- 118
VAMP-1_RAT          63  SELDDRADALQAGASVFESSAAKLKRKYWWKNCKMMIMLGAICAIIVVVIVIYIFT- 118
VAMP-1_CHICKEN      64  SELDDRADALQAGASVFESSAAKLKRKYWWKNCKMMIMMGVICAIVVVVIVIYFFT- 119
VAMP-1_MOUSE        63  SELDDRADALQAGASQFESSAAKLKRKYWWKNCKMMIMLGAICAIIVVVIVIYFFT- 118
VAMP-1_G.PIG        63  SELDDRADALQAGASQFESSAAKLKRKYWWKNCKMMIMLGAICAIIVVVIVIYFFT- 118
VAMP-1_C.ELEGANS    46  SQLDDRADALQEGASQFEKSAATLKRKYWWKNIKMMIIMCAIVVILIIIIVLWAGGK 109
VAMP-1_XENOPUS      53  SELDDRADALQAGASQFETSAAKLKRKYWWKNCKMWAILIAVVLVIIIIIIVWSVS- 118
                        *:********* *** **.***.********* **  ::  .:   ::::*:::

VAMP-2_HUMAN        1   MSATAATAPPAAPAGEGGPPAPPPNLTSNRRLQQTQAQVDEVVDIMRVNVDKVLERDQKL 60
VAMP-2_RAT          1   MSATAATVPPAAPAGEGGPPAPPPNLTSNRRLQQTQAQVDEVVDIMRVNVDKVLERDQKL 60
VAMP-2_CHICKEN      1   MSAPAPTQGPTST-G-AAGPPPATNVSSNKRLQQTQAQVDEVVDIMRMNVDKVLERDQKL 58
VAMP-2_MOUSE        1   MSATAATVPPAAPAGEGGPPAPPPNLTSNRRLQQTQAQVDEVVDIMRVNVDKVLERDQKL 60
VAMP-2_G.PIG        1   RSATAATAPPAAPAGEGGPPAPPPNLTSNRRLQQTQAQVDEVVDIMRVNVDKVLERDQKL 60
VAMP-2_DROSOPHILA   1   -----MSAPAGAPAPEGGNQAPP-NLTSNRRLQQTQAQVDEVVDIMRVNVDKVLERDQKL 60
VAMP-2_XENOPUS      1   --MSAPAAGPPAAAPGDGAPQGPPNLTSNRRLQQTQAQVDEVVDIMRVNVDKVLERDTKL 60
                              :. . *.*  .*    * *::**:*****************:********* **

VAMP-2_HUMAN        61  SELDDRADALQAGASQFETSAAKLKRKYWWKNLKMMIILGVICAIILIIIIVYFST 116
VAMP-2_RAT          61  SELDDRADALQAGASQFETSAAKLKRKYWWKNLKMMIILGVICAIILIIIIVYFST 116
VAMP-2_CHICKEN      59  SELDNRADALQAGASQFETSAAKLKRKYWWKNCKMMIILGVVCTVILIIIIIYFST 114
VAMP-2_MOUSE        61  SELDDRADALQAGASQFETSAAKLKRKYWWKNLKMMIILGVICAIILIIIIVYFST 116
VAMP-2_G.PIG        61  SELDDRADALQAGASQFETSAAKLKRKYWWKNLKMMIILGVICAIILIIIIVYFST 116
VAMP-2_DROSOPHILA   55  SELDDRADALQAGASQFETSAAKLKNKYWWKNAKMMIILGVICVIVLIIIIVYFST 111
VAMP-2_XENOPUS      59  SELDDRADALQAGASQFETSAAKLKRKYWWKNMKMMIIMGVICAIILIIIIVYFST 114
                        ****:********************.****** *****:****.*:*****:****
```

Supplementary Figure S1. Alignment of VAMP-1 (top) and VAMP-2 showing that the two proteins are highly conserved FETSAAKLKRKYW peptide (pink) generated by BoNT/B and TeNT is present in all the main animal species used in research. C) Scheme showing the generation of the immunogenic carrier by chemical conjugation of the C-terminal Cysteine to the Keyhole limpet Hemocyanin. D) the peptide-KLH complex was injected into a rabbit and at the scheduled time points the blood was collected for the ensuing purification of peptide-specific IgGs.

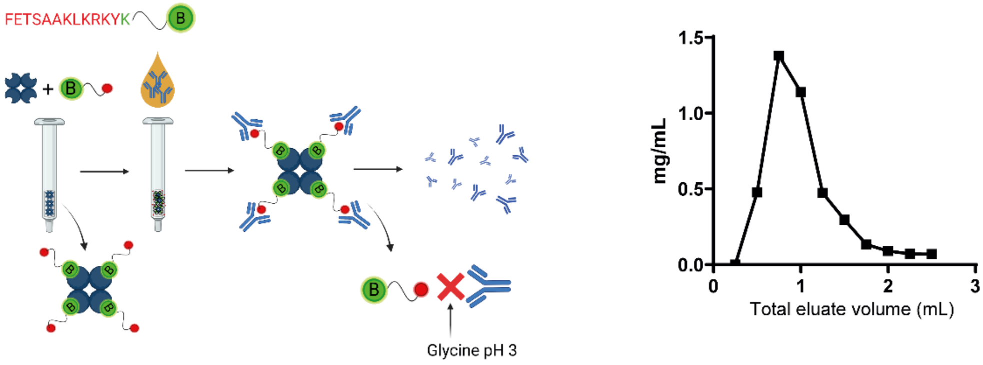

Supplementary Figure S2. Purification strategy of the $VAMP_{77}$ polyclonal antibody. The left panel shows the antigenic peptide conjugated to a biotin residue for the fast coupling to the streptavidin-agarose resin. The streptavidin-peptide complex was then packed inside disposable columns and the immune serum was incubated overnight for antigen binding. Antibodies were then eluted with sequential additions of Glycine solution at pH 3, which induces the release of bound IgG without affecting the biotin-streptavidin interaction. On the right is reported the chromatogram of the IgG fractions obtained during the elution.

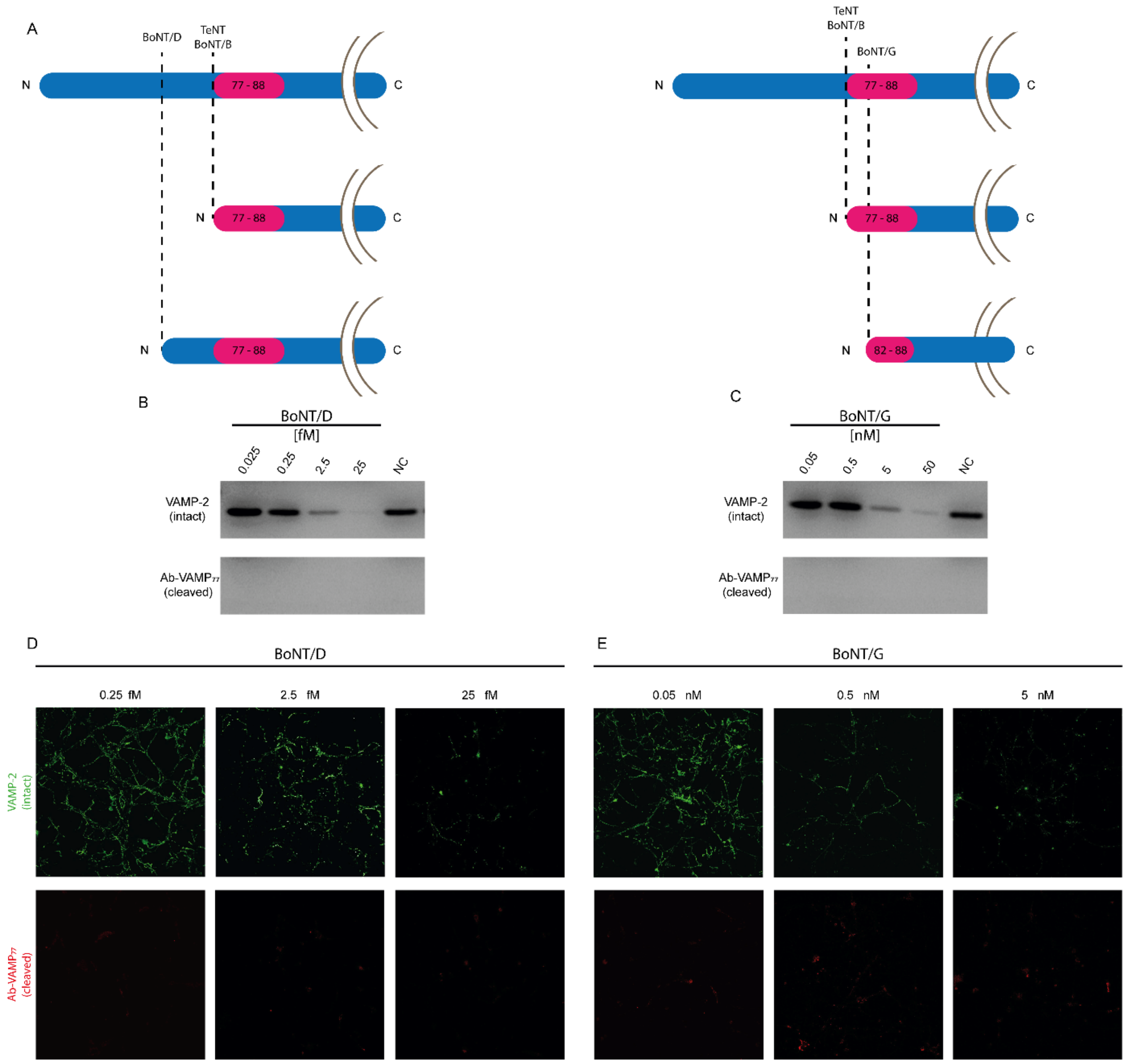

Supplementary Figure S3. A) Scheme showing the different fragments generated in VAMP-2 after the cleavage by BoNT/D, TeNT, BoNT/B, and BoNT/G, highlighting the sequence corresponding to the antigenic peptide used to generate the $VAMP_{77}$ antibody. B) and C) Representative western blotting showing that the $VAMP_{77}$ antibody does not recognize VAMP truncated by BoNT/D and BoNT/G in CGNs treated with the indicated concentrations of toxins. The NC samples are control neurons not treated with any toxin. C) and D) show the same experiment evaluated by imaging using the same antibodies.

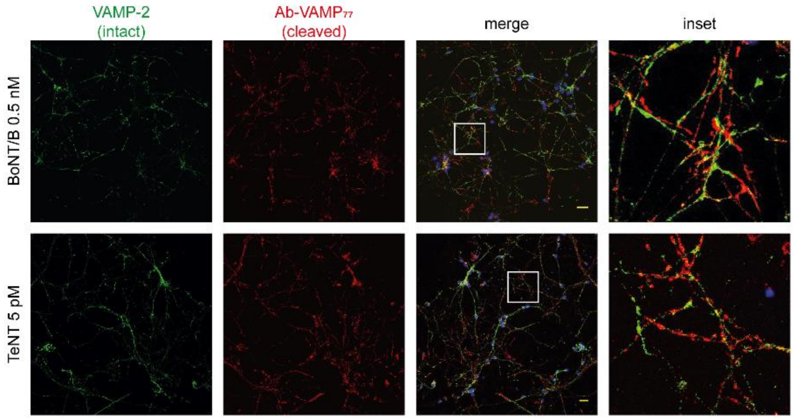

Supplementary Figure S4. Immunofluorescence analysis shows that in CGNs treated with TeNT and BoNT/B at the indicated concentrations, the antibody against VAMP-2 and the VAMP77 monoclonal antibody do recognize two different populations of VAMP, i.e., the intact and the cleaved form.

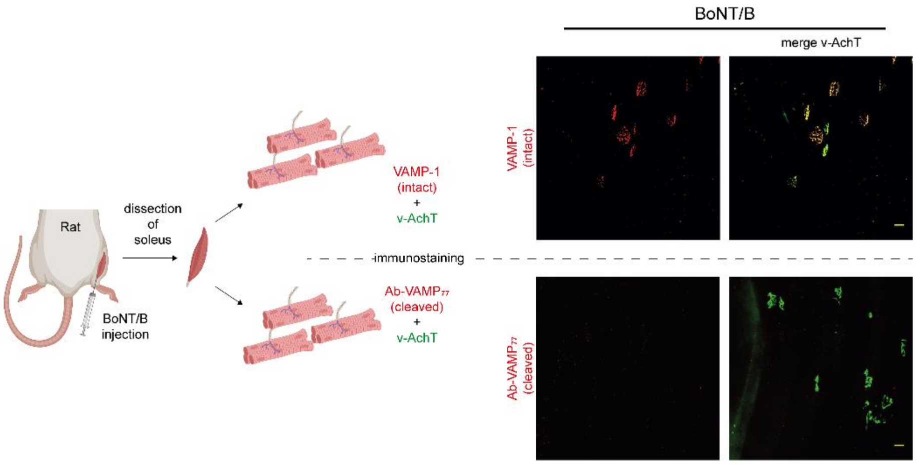

Supplementary Figure S5. BoNT/B does not cleave VAMP at the NMJ of the rat. A) Scheme showing the injection of BoNT/B in the rat hind limb. After 48 hours, the soleus muscle was dissected and fixed. Muscle bundles of about 20-30 myofibers were then stained with an antibody either against intact VAMP-1 (red in top panels) or with the VAMP77 antibody (red in bottom panels) to assess VAMP cleavage. In both conditions, the muscle was stained with an antibody against the v-AchT to spot the NMJs.
